# Delta Opioid Receptors: The Link between Exercise and Cardioprotection

**DOI:** 10.1371/journal.pone.0113541

**Published:** 2014-11-21

**Authors:** Juliana P. Borges, Karine S. Verdoorn, Anissa Daliry, Scott K. Powers, Victor H. Ortenzi, Rodrigo S. Fortunato, Eduardo Tibiriçá, Marcos Adriano Lessa

**Affiliations:** 1 Laboratory of Cardiovascular Investigation, Oswaldo Cruz Institute, FIOCRUZ, Rio de Janeiro, RJ, Brazil; 2 Federal University of Rio de Janeiro, Macaé, RJ, Brazil; 3 Department of Applied Physiology and Kinesiology, University of Florida, Gainesville, FL, United States of America; 4 Carlos Chagas Filho Biophysics Institute, Federal University of Rio de Janeiro, Rio de Janeiro, RJ, Brazil; Emory University, United States of America

## Abstract

This study investigated the role of opioid receptor (OR) subtypes as a mechanism by which endurance exercise promotes cardioprotection against myocardial ischemia-reperfusion (IR) injury. Wistar rats were randomly divided into one of seven experimental groups: 1) control; 2) exercise-trained; 3) exercise-trained plus a non-selective OR antagonist; 4) control sham; 5) exercise-trained plus a kappa OR antagonist; 6) exercise-trained plus a delta OR antagonist; and 7) exercise-trained plus a mu OR antagonist. The exercised animals underwent 4 consecutive days of treadmill training (60 min/day at ∼70% of maximal oxygen consumption). All groups except the sham group were exposed to an in vivo myocardial IR insult, and the myocardial infarct size (IS) was determined histologically. Myocardial capillary density, OR subtype expression, heat shock protein 72 (HSP72) expression, and antioxidant enzyme activity were measured in the hearts of both the exercised and control groups. Exercise training significantly reduced the myocardial IS by approximately 34%. Pharmacological blockade of the kappa or mu OR subtypes did not blunt exercise-induced cardioprotection against IR-mediated infarction, whereas treatment of animals with a non-selective OR antagonist or a delta OR antagonist abolished exercise-induced cardioprotection. Exercise training enhanced the activities of myocardial superoxide dismutase (SOD) and catalase but did not increase the left ventricular capillary density or the mRNA levels of HSP72, SOD, and catalase. In addition, exercise significantly reduced the protein expression of kappa and delta ORs in the heart by 44% and 37%, respectively. Together, these results indicate that ORs contribute to the cardioprotection conferred by endurance exercise, with the delta OR subtype playing a key role in this response.

## Introduction

The concept that regular exercise is cardioprotective against ischemia-reperfusion (IR) cardiac injury is well established in animal models [1], [2], and human epidemiological studies also suggest that physically active individuals are protected against IR-induced myocardial injury [3]–[5]. Indeed, physically active individuals have a lower incidence of myocardial infarction and a greater survival rate following a heart attack compared with their less active counterparts [1], [3].

In reference to animal investigations of cardioprotection, well-controlled animal studies beginning in the late 1970s have provided convincing evidence that regular bouts of endurance exercise provide cardioprotection against IR-induced injury [1], [2]. Although it is clear that endurance exercise produces a cardioprotective phenotype, the mechanisms responsible for this phenomenon remain unclear. Some studies suggest that exercise promotes cardioprotection, at least in part, by direct effects on the myocardium [1], [2], [6]. Specific mechanisms that may be responsible for exercise-induced cardioprotection include the following: (a) increased nitric oxide production and cardiac antioxidant capacity [2], [7]–[11]; (b) expansion of the coronary capillary network and enlargement of the coronary artery diameter [2], [7]; (c) increased production of heat shock proteins (HSP) [2], [7], [8]; (d) reduced production of reactive oxygen species (ROS) in myocardial mitochondria during IR [7], [12]; and (e) improved function of sarcolemmal and/or mitochondrial ATP-sensitive potassium channels (sarco/mito K+ ATP channels) [7], [8].

Additionally, recent evidence suggests that endogenous opioids may contribute to exercise-induced cardioprotection [13], [14]. Dickson et al. [13] demonstrated that the exercise-induced reduction in infarct size (IS) was abolished following the blockade of opioid receptors (OR) with naloxone. Similarly, Michelsen et al. [14] showed that naloxone treatment blocked the exercise-induced cardioprotection transferred from a remotely preconditioned human donor to an isolated perfused rabbit heart using a dialysate of plasma.

Identical to exercise-induced cardioprotection, ischemic preconditioning (IPC), which consists of performing intermittent brief exposures of the myocardium to ischemia, protects the heart against a subsequent severe episode of ischemia [15]. Strong evidence also indicates that endogenous opioids are involved in cardioprotection induced by IPC [14], [16]–[20]. Intriguingly, despite the putative role of the opioid system as a mechanism for IPC, the involvement of different subtypes of ORs in the cardioprotective effect of exercise is not completely understood. To date, few studies have investigated the role that OR activation plays in exercise-induced cardioprotection [13], [21]. Therefore, the current experiments were designed to identify the specific OR subtype responsible for exercise-induced cardioprotection. Based upon our preliminary experiments, we hypothesized that the delta OR subtype plays an essential role in exercise-induced cardioprotection.

## Materials and Methods

### Animals and ethics statement

Male Wistar rats (250–300 g) were housed under controlled light (12:12 h light-dark cycle) and temperature (22±1°C) conditions with free access to water and standard rat chow.

This study was carried out in strict accordance with the Guidelines for the Care and Use of Laboratory Animals of the National Institutes of Health. The Oswaldo Cruz Foundation Animal Welfare Committee approved all protocols (permit number: LW-4/11). All surgeries were performed under sodium pentobarbital anesthesia, and all efforts were made to minimize animal suffering.

### Experimental design

**Figure 1:**
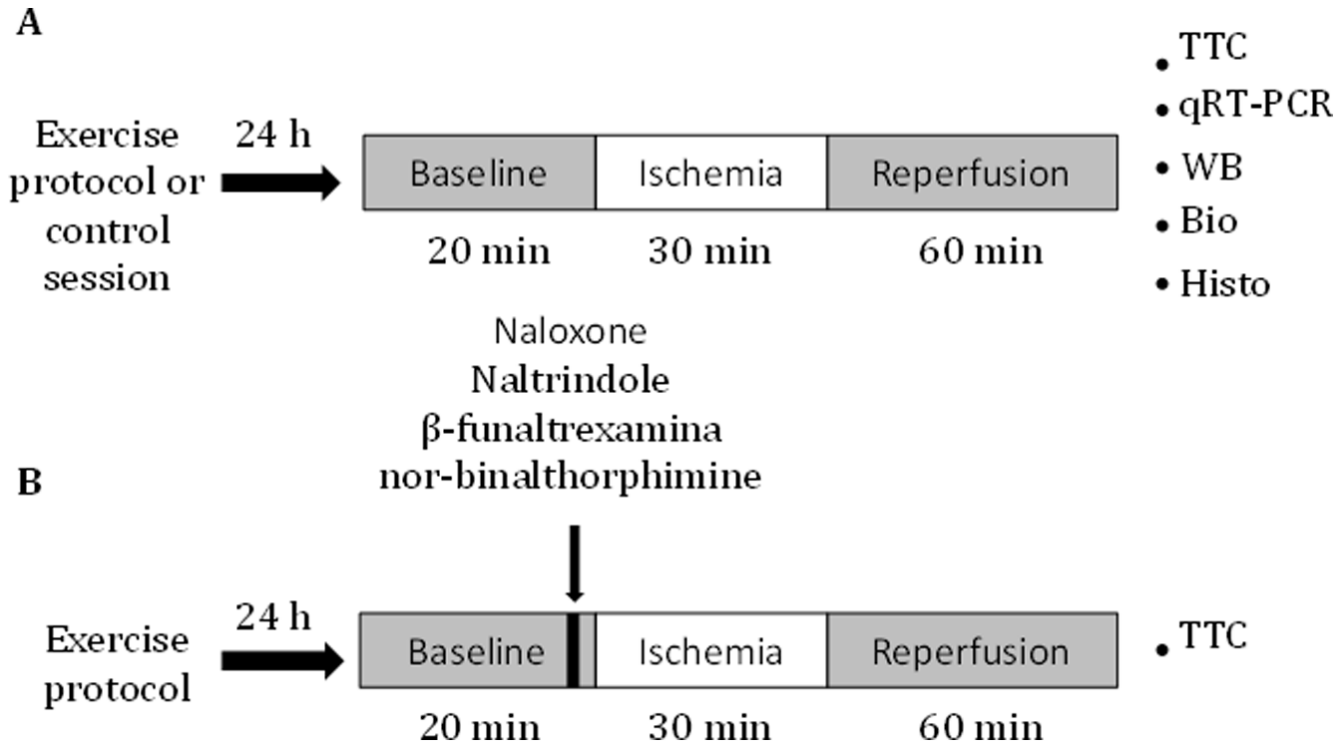
Diagrammatic representation of the experimental design of the study. (A) Left ventricles obtained from the exercised and control groups were submitted to qRT-PCR, Western blotting (WB), histochemical (Histo), and biochemical (Bio) analyses and TTC staining. (B) Left ventricles obtained from the non-selective (naloxone) and selective OR antagonist (naltrindole, β-funaltrexamine and nor-binalthorphimine) groups were submitted to TTC staining.

These experiments evaluated whether the delta OR subtype plays an essential role in exercise-induced cardioprotection. The experimental groups were subjected to endurance exercise training and treated with both non-selective and selective OR antagonists prior to exposure to a myocardial IR insult. The rats were randomly divided into one of seven experimental groups: 1) control (CT; n = 20); 2) exercised (exe; n = 18); 3) exercised plus treatment with a non-selective OR antagonist (Exe+NAL; n = 10); 4) control sham (Sham; n = 10); 5) exercised plus treatment with the kappa OR (KOR) antagonist (Exe+KOR; n = 5); 6) exercised plus treatment with the delta OR (DOR) antagonist (Exe+DOR; n = 5); and 7) exercised plus treatment with a mu OR (MOR) antagonist (Exe+MOR; n = 5). All of the groups were exposed to IR injury surgery (see below), with the exception of the sham group, which was submitted to sham injury surgery without exposure to IR. Following reperfusion, the left ventricles were excised to histologically quantify the IS and assess a variety of biochemical measurements (Figure 1).

### Opioid receptor (OR) blockade

To identify which of the three OR subtypes are involved in exercise-induced cardioprotection, 10 min prior to the induction of myocardial ischemia, select groups of animals received an intravenous injection of a selective OR antagonist. Specifically, the group of animals assigned to the DOR antagonist received 3 mg/kg of naltrindole. Similarly, animals assigned to MOR blockade were treated with 3 mg/kg of β-funaltrexamine, and animals assigned to KOR blockade were injected with 3 mg/kg of nor-binalthorphimine. Finally, animals assigned to the non-selective OR group received 3 mg/kg of naloxone to block all OR receptor subtypes. The 3 mg/kg dose of OR antagonists was chosen based on previous results from Schultz et al. [17], [44] and on preliminary experiments from our lab.

### Endurance exercise training

The rats were familiarized to treadmill running using a low-speed, motor-driven rodent treadmill (HT 2.0, Hectron Fitness Equipment, RJ, Brazil). The animals began by walking at 12 m/min (0% grade) for 15 min/day on three consecutive days. Following this brief period of familiarization, maximal oxygen consumption (VO_2max_) was measured during the last stage of a graded treadmill exercise test. The graded exercise protocol began with the rats running at 10 m/min (0% grade) with the treadmill speed increasing by 3 m/min every 3 min until the animals could no longer maintain the desired running speed. VO_2max_ was measured by assessing the total airflow through the treadmill chamber and assessing the oxygen content of the expired gas using an electronic oxygen analyzer (AVS Projetos, SP, Brazil). The measurement of VO_2max_ was then used to establish the appropriate treadmill speed to elicit a relative exercise intensity of ∼70% VO_2max_.

Following the VO_2max_ test, the animals completed an additional four consecutive days of exercise training (60 min/day, 0% grade, at ∼70% of VO_2max_). This exercise training protocol was selected because this training program has been shown to promote cardioprotection against IR injury [21]. The exercise training occurred between 8:00 and 10:00 am each day. To control for handling/environmental stress between experimental groups, the animals assigned to the sedentary groups were placed on a non-moving treadmill for 60 minutes. Twenty-four hours after the last exercise or control session, the animals were exposed to IR injury surgery.

### Surgical procedures and infarct area assessment

Anesthesia was induced and maintained with sodium pentobarbital (70 mg/kg intraperitoneally (ip) and 5 mg/kg intravenously (iv), respectively), and the animals were tracheotomized and artificially ventilated (Rodent Ventilator 7025, Ugo Basile, Varese, Italy) with a respiratory frequency of 50 breaths/min. Polyethylene-tipped cannulas filled with heparinized saline solution were inserted into the left femoral artery and the right jugular vein for direct measurements of arterial pressure and drug administration, respectively. The arterial cannula was connected to a strain-gauge transducer (TSD104A, BIOPAC Systems, CA, USA), and pressure signals were channeled through a signal amplifier (DA100C, BIOPAC Systems, CA, USA). Data were acquired at a rate of 1,000 samples/s through the MP150 system (BIOPAC Systems, CA, USA) and recorded using AcqKnowledge 3.7.3 for Windows (BIOPAC Systems, CA, USA).

The animals were submitted to left thoracotomy between the fourth and fifth ribs, and after 20 min of baseline, myocardial IR injury was induced by the occlusion of the left anterior descending coronary artery for 30 min, followed by reperfusion for 60 min. The sham group underwent the same surgical procedure without ischemia. To distinguish necrotic from viable myocardium, the left ventricles were submitted to a gross histological double-staining technique using the Evan's blue/triphenyl tetrazolium chloride (TTC) method as previously described [13]. The myocardial infarct area was assessed in each heart by a blinded observer using planimetry (Image J, NIH Image, USA), and the infarct area was expressed as a percentage of the area at risk (% AAR).

### Analysis of cardiac capillary density

To measure the left ventricular structural capillary density, the samples were dehydrated in a graded series of ethanol (70%, 95%, and 100%) and cut using the “orthrip” method [22]. This method generates isotropic, uniform, and random sections of biological specimens for the quantitative assessment of three-dimensional anisotropic structures (such as the left ventricle) from two-dimensional sections. Therefore, the organ was cut three times consecutively. The first section was random, the second section was orthogonal to the first, and the third section was orthogonal to the second [23]. After the samples were embedded in paraffin blocks, they were cut into 5-µm-thick sections and stained with FITC-conjugated *G. simplicifolia lectin* (1∶150 dilutions) in a dark chamber at room temperature for 30 min. For each rat, at least five randomly selected microscopic fields were examined by microscopy (Olympus BX51/WI; Olympus, PA, USA) and analyzed with Archimed 3.7.0 software (Microvision, Lisses, France). The left ventricular structural capillary density was calculated by dividing the capillary volume density by the fiber volume density (Vv_[cap]_/Vv_[fib]_).

### Determination of antioxidant enzyme activities

Samples of frozen left ventricles were homogenized with an Ultra-Turrax (Janke & Kunkel IKA, Labortechnik, Germany) in nine volumes of 10 mM Tris–HCl pH 7.4 containing 0.9% NaCl, 1 mM PMSF and 0.5 g/ml aprotinin. The homogenate was centrifuged at 720×g at 4°C for 10 min, and the supernatant was utilized for enzymatic measurements [24], [25]. Protein content was determined by the Bradford method using bovine serum albumin as the standard [26]. All enzyme activity assays were performed at 37°C with at least four biological replicates for the Exe and CT groups. Readings were obtained using a GBC 920 UV–Vis spectrophotometer (GBC Scientific Equipment, Australia), and the results were expressed as units (mol/min) per mg of protein. The total superoxide dismutase (SOD) activity was determined according to a method described elsewhere [27]. Briefly, the total SOD activity was assessed by the rate of cytochrome c reduction at 550 nm before and after adding the heart homogenate (15 µg protein) in a reaction medium containing 50 mM phosphate buffer (pH 8.0), 0.1 mM EDTA, 0.01 mM potassium cyanide, 0.02 mM cytochrome c, 0.05 mM xanthine, and 8 mU xanthine oxidase. A 50% decrease in the rate of cytochrome c reduction was considered as one international unit (IU) of total SOD activity and was expressed as IUs per mg of protein. Catalase activity was measured by the rate of hydrogen peroxide (H_2_O_2_) decomposition to water and oxygen, according to a previous method reported in the literature [28]. The difference between the rate of absorbance decrease at 240 nm in the presence or absence of heart homogenates (30 µg protein) was measured in a reaction medium composed of 50 mM phosphate buffer (pH 7.0), 0.002% Triton X-100, 0.1 mM EDTA, and 15 mM H_2_O_2_ in a final volume of 1 ml. The amount of H_2_O_2_ was calculated using the molar extinction coefficient (43.6 M/cm), and catalase activity was expressed in units (µmol of H_2_O_2_ consumed per min) by mg protein.

### Quantitative RT-PCR

Total RNA was extracted from 30 mg of at least four samples of frozen left ventricles obtained from the CT and Exe groups using the RNeasy Fibrous Tissue Mini kit (Qiagen, Hilden, Germany), following the manufacturer's instructions. The RNA yield and purity were determined using a NanoDrop spectrometer (Thermo Fisher Scientific, MA, USA). Next, 1 µg of total RNA was reverse-transcribed into cDNA using the High Capacity cDNA Reverse Transcriptase kit (Applied Biosystems, CA, USA) according to the manufacturer's instructions. The amplification reactions were performed in 96-well plates (Applied Biosystems, CA, USA) at a final volume of 25 µl and contained 1 µl of 10× diluted cDNA, 12.5 µl of 2× Power SYBR Green PCR Master Mix (Applied Biosystems, CA, USA) and 150 nM of each forward and reverse primer (Thermo Fisher Scientific, MA, USA) (Table 1). The levels of mRNA were measured using the ViiA 7 Real Time PCR System (Thermo Fisher Scientific, MA, USA). The amplification program consisted of an initial cycle at 50°C for 2 min, followed by 95°C for 10 min, and 40 cycles of 95°C for 15 s and 60°C for 1 min. Each cDNA was amplified in duplicate, and a corresponding sample without reverse transcriptase (no-RT sample) was included as a negative control. The specificity of the RT-PCR products was confirmed by melting curve analysis and by omission of the reverse transcriptase. All of the samples were normalized to β-actin mRNA using the ΔΔCt method.

**Table 1:** Primer sequences.

*Target*	*Primer Sequence*	
EcSOD	Forward	GGCCCAGCTCCAGACTGA
	Reverse	CTCAGGTCCCCGAACTCATG
CuZnSOD	Forward	CGGCTTCTGTCGTCTCCT
	Reverse	GTTCACCGCTTGCCTTCT
MnSOD	Forward	TTAACGCGCAGATCATGCA
	Reverse	CCTCGGTGACGTTCAGATTGT
Catalase	Forward	ACTCAGGTGCGGACATTC
	Reverse	GGAGTTGTACTGGTCCAGAAGAGCC
HSP72	Forward	GCTCATCAAGCGCAACTCCAC
	Reverse	TCGTACACCTGGATCAGCACCC
β-Actin	Forward	CCACCCGCGAGTACAACCTTCTT
	Reverse	GAAGCCGGCCTTGCACATGCC

EcSOD, endothelial cell superoxide dismutase (SOD); CuZnSOD, copper zinc SOD; MnSOD, manganese SOD; HSP72, heat shock protein 72.

### Western blotting

Samples of frozen left ventricles from the Exe and CT groups were minced on ice and homogenized with a RW20 tissue processor (Digital IKA, Staufen, Germany) for 2 min at 1,745 rpm in homogenization buffer containing 250 mM sucrose, 1 mM imidazole, pH 7.6 (adjusted with Tris), 1 mM EDTA and 0.05 g protease inhibitor. The homogenates were centrifuged at 3,500 rpm for 15 min (SS34 rotor, SORVALL RC-5B centrifuge, Thermo Fisher Scientific, MA, USA), and the supernatant was collected, centrifuged again at 32,900 rpm for 60 min (70 Ti rotor, Beckman Coulter centrifuge, Beckman Coulter, CA, USA) (modified from Dostanic et al., 2004) [29], and kept on ice. Then, a 10-µl aliquot (in triplicate) was used for the protein concentration assays, which were performed using the Folin phenol method described by Lowry et al. [30] with 5% SDS added to the samples and bovine serum albumin used as the standard. Sixty micrograms of protein were separated by 10% polyacrylamide gel electrophoresis and subsequently electroblotted onto nitrocellulose membranes (Amersham GE Healthcare, Buckinghamshire, United Kingdom). The resulting membranes were then stained with Ponceau S and analyzed to verify equal loading and transfer. The membranes were blocked overnight with 5% skim milk at 4°C in phosphate-buffered saline solution containing Tris-buffered saline (TBS). The blots were then incubated in blocking buffer with rabbit polyclonal IgG antibodies directed against OR subtypes mu (1∶1,000, Santa Cruz Biotechnology, SC-15310, CA, USA), kappa (1∶1,000, Santa Cruz Biotechnology, SC-9112, CA, USA) and delta (1∶1,500, Santa Cruz Biotechnology, SC- 9111, CA, USA) for 1 h at room temperature. After washing with TBS-T (TBS with 0.1% Tween 20), the blots were incubated at room temperature for 1 h with the appropriate secondary polyclonal antibody (anti-rabbit 1∶5,000, Amersham GE Healthcare, Buckinghamshire, United Kingdom) coupled to peroxidase and washed again with TBS-T. The membranes were then treated with chemiluminescent reagents (luminol and enhancer; Amersham GE Healthcare, Buckinghamshire, United Kingdom) and exposed to light-sensitive film. Images of these films were analyzed using Scion software (Scion Co, MD, USA). The results were expressed in arbitrary units (a.u.).

### Reagent sources

All drugs and reagents were purchased from Sigma Chemical (MO, USA), with the exception of naloxone, naltrindole, β-funaltrexamine, and nor-binalthorphimine (Tocris Bioscience, MN, USA).

### Statistical analysis

Statistical comparisons between groups were performed using a one-way analysis of variance. Hemodynamic parameters were evaluated using a two-way analysis of variance. The Bonferroni post-test was used to localize the significant differences. P values <0.05 were considered statistically significant. The results are expressed as the mean ± SD.

## Results

### Hemodynamic parameters

The baseline hemodynamic parameters were not significantly different between experimental groups. Not surprisingly, ischemia produced a significant decrease in systolic, mean and diastolic arterial pressure 5 min after coronary ligation in all groups compared with the sham group (Table 2).

**Table 2:** Hemodynamic parameters in response to IR injury.

		Baseline	5 min after ischemia	30 min after ischemia	60 min after reperfusion
**CT**	**SAP**	126±12	84±17	96±16	103±25
	**MAP**	102±11	67±18	83±18	83±24
	**DAP**	82±12	54±16	68±18	66±21
	**HR**	428±59	451±24	456±36	416±73
**Exe**	**SAP**	122±14	84±19	124±29	119±11
	**MAP**	105±12	69±18	105±29	97±11
	**DAP**	89±12	58±16	89±27	77±10
	**HR**	449±34	454±27	473±26	462±31
**Exe + Nal**	**SAP**	124±19	90±29	109±27	124±23
	**MAP**	106±16	76±27	90±27	101±21
	**DAP**	88±13	65±26	74±26	83±18
	**HR**	452±32	465±43	471±34	463±37
**Sham**	**SAP**	118±7	119±9*	121±13	128±7
	**MAP**	101±7	101±9*	100±13	103±9
	**DAP**	87±7	85±9*	81±12	81±10
	**HR**	452±39	455±36	445±33	441±45

The values represent the means ± SD, n = 10 for all groups. CT, control group; Exe, exercised group; Exe+Nal, exercised + naloxone group; SAP, systolic arterial pressure (mmHg); MAP, mean arterial pressure (mmHg); DAP, diastolic arterial pressure (mmHg); HR, heart rate (bpm).

*P<0.05 *vs.* all groups.

### Infarct size (IS)

**Figure 2:**
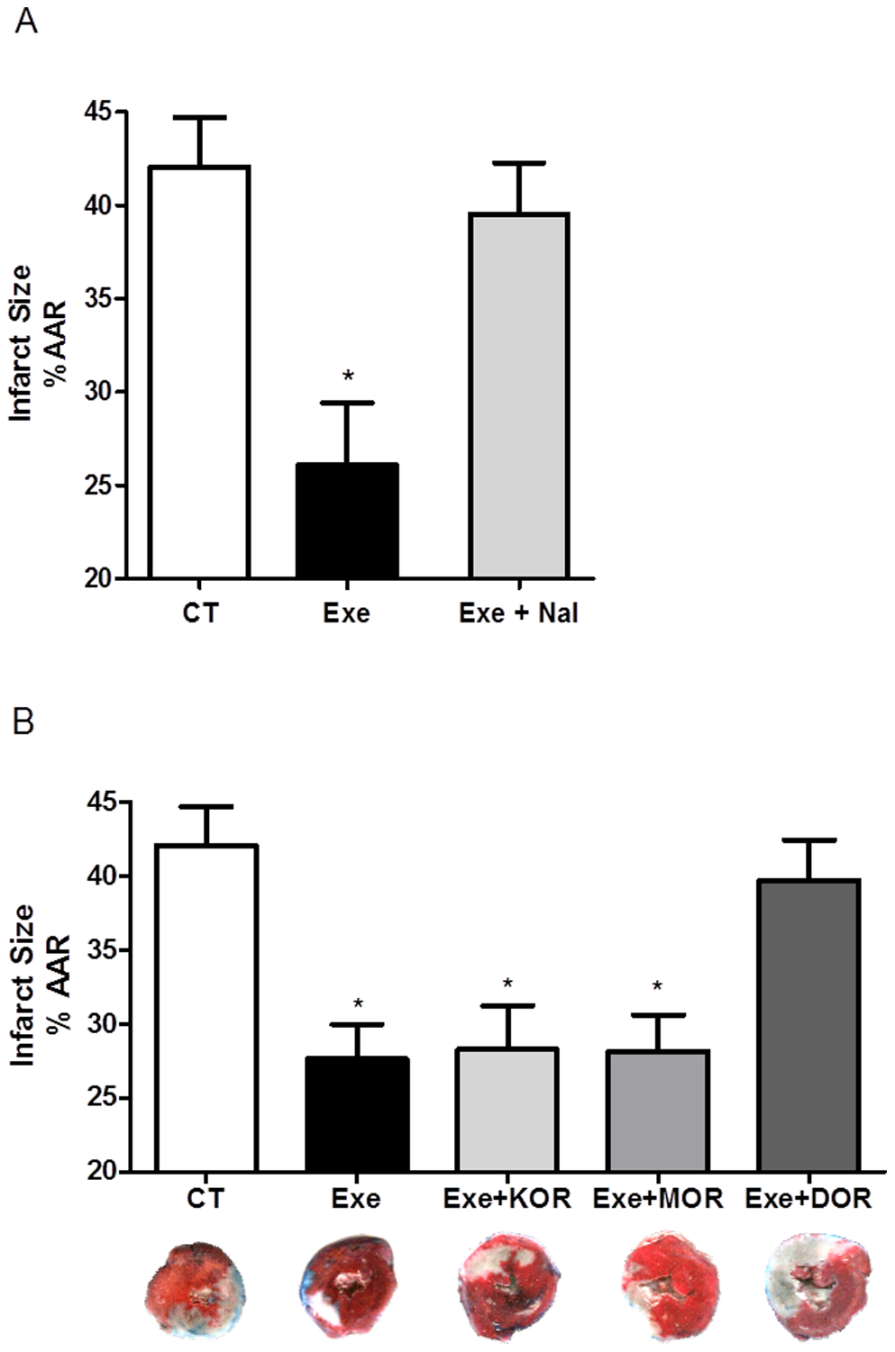
IS after ischemia-reperfusion injury following non-selective (A) and selective (B) opioid receptor antagonists. CT (control group, n = 10); Exe (exercised group, n = 10); Exe+Nal (non-specific antagonist group, n = 10); Exe+KOR (kappa opioid receptor antagonist group, n = 5); Exe+MOR (mu opioid receptor antagonist group, n = 5); and Exe+DOR (delta opioid receptor antagonist group, n = 5). * P <0.05 *vs.* CT.

As expected, the sham group experienced no myocardial infarction in response to the sham surgery. Importantly, the Exe group showed a significantly reduced IS compared with the CT group (27.6±3.5 *vs.* 42.0±3.0%; P<0.05). This cardioprotection was abolished when ORs were blocked with naloxone, maintaining an infarct area similar to that of the CT group (Exe+Nal: 39.5±2.9%; P<0.05 - Figure 2A).

To determine which OR mediated this cardioprotective effect, specific receptor subtype antagonists were used. MOR and KOR blockade did not interfere in the exercise-induced cardioprotection, these groups presented IS similar to Exe group (Exe+MOR: 28.1±2.7%; Exe+KOR: 28.2±4.8% - Figure 2B). Nevertheless, DOR blockade completely prevented the cardioprotection seen in the Exe group, the IS was similar to that observed in the CT group (Exe+DOR: 39.6±2.0% - Figure 2B).

### Left ventricle structural capillary density

**Figure 3:**
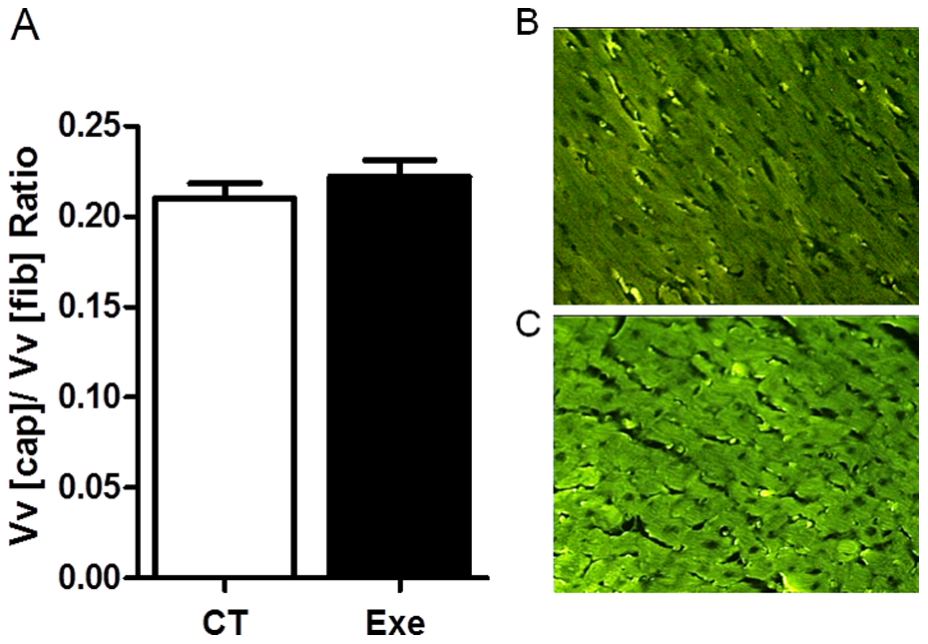
Left ventricle structural capillary density. The bars represent capillary density in the control and exercised groups (A). The representative images used to determine the structural capillary density with capillaries stained with FITC-conjugated *G*. *simplicifolia* lectin. Magnification, ×200; scale bar, 100 µm in the CT (B) and Exe groups (C). Exe, exercised group (n = 5); CT, control group (n = 5).

The left ventricle structural capillary density was similar in both the Exe (0.22±0.01 Vv_[cap]_/Vv_[fib]_) and CT groups (0.21±0.01 Vv_[cap]_/Vv_[fib]_ - Figure 3).

### Antioxidant enzyme activities

**Figure 4:**
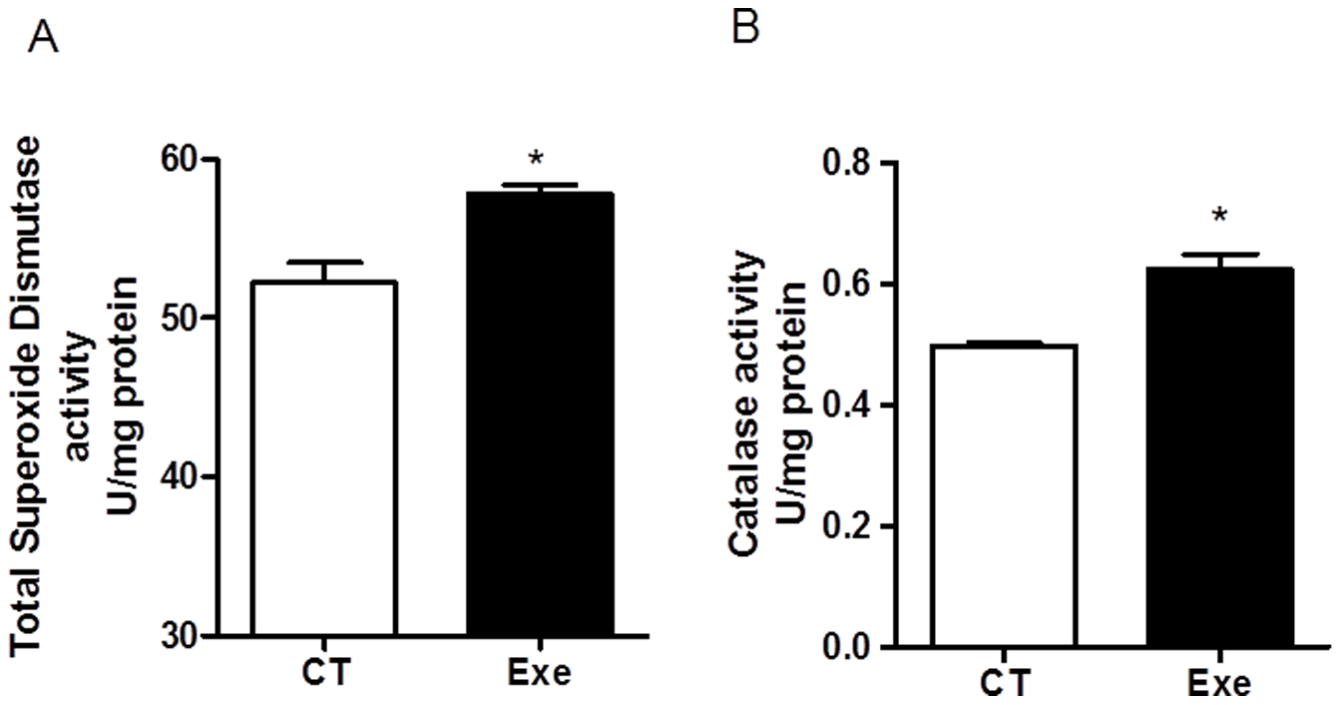
Total superoxide dismutase (A) and catalase (B) activities in the control and exercised groups. Exe, exercised group (n = 5); CT, control group (n = 5). * P<0.05 *vs.* CT.

Exercise caused a significant increase in the total SOD (CT: 52.2±2.2 *vs.* Exe: 57.8±1.2 U/mg; P<0.05) and catalase activities (CT: 0.49±0.00 *vs.* exe: 0.62±0.05 U/mg; P<0.05 - Figure 4).

### HSP72 and antioxidant enzyme mRNA expression

**Figure 5:**
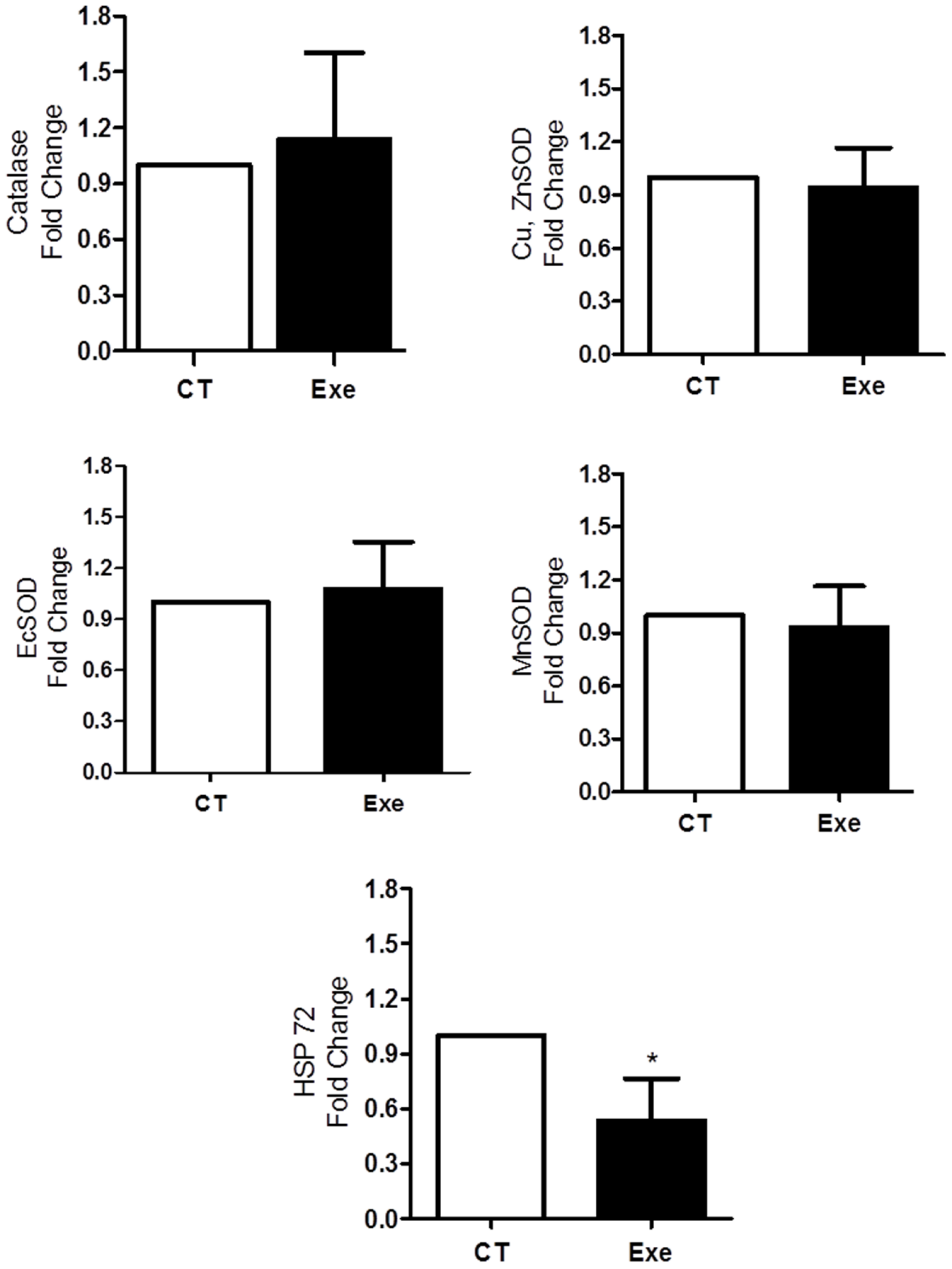
mRNA expression of key antioxidant enzymes and HSP72. Copper zinc superoxide dismutase (SOD) (CuZnSOD), endothelial cell SOD (EcSOD), manganese SOD (MnSOD) and heat shock protein 72 (HSP72). The values represent control fold changes, normalized by β-actin. Exe (exercised group, n = 3); and CT (control group, n = 5). * P<0.05 *vs.* CT.

In the mRNA expression measurements of antioxidant enzymes, the endothelial cell SOD (EcSOD), copper zinc SOD (CuZnSOD), manganese SOD (MnSOD) and catalase expression levels revealed no differences between the Exe and CT groups. For each enzyme, the fold change in expression relative to the control was 1.07±0.86, 0.94±0.18, 0.93±0.19 and 1.13±0.38, respectively (Figure 5). Whereas exercise induced no adaptations in any antioxidant enzyme expression, HSP72 mRNA expression in the Exe group was significantly lower compared with the CT group (0.53±0.18-fold control; P<0.05 - Figure 5).

### OR protein expression

**Figure 6:**
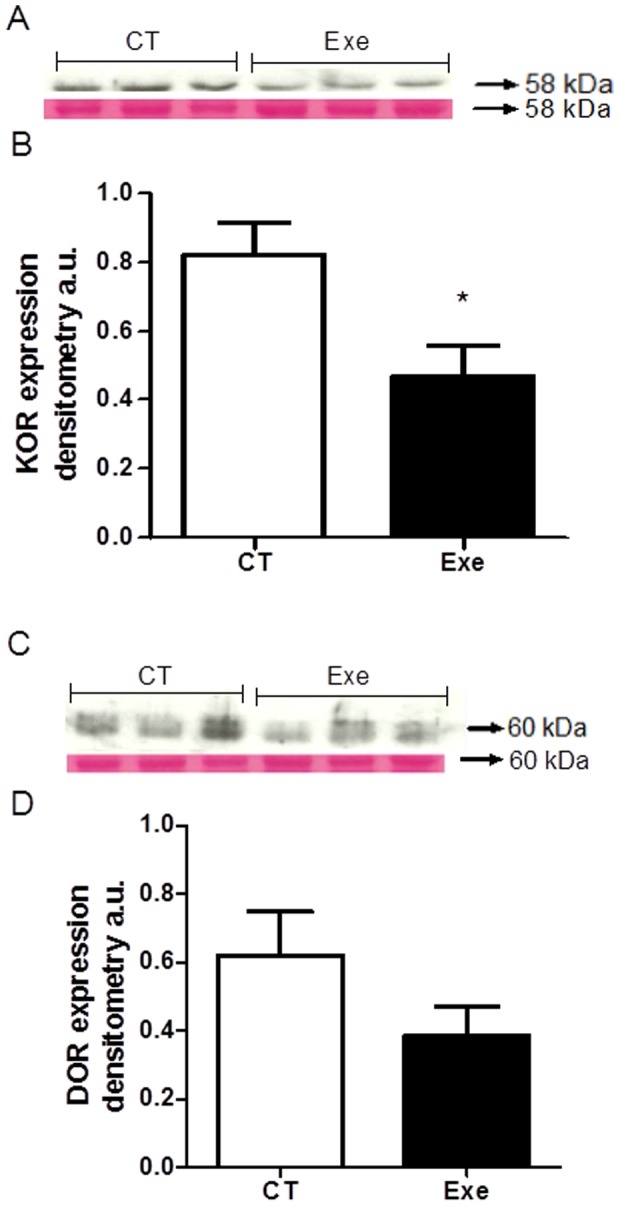
OR subtype expression after IR injury. Immunoblots of the control (CT; n = 4) and exercised (Exe; n = 5) group bands for the kappa opioid receptor (KOR) (A) and delta opioid receptor (DOR) (C). The bars represent the KOR (B) and DOR (D) expression levels normalized by *Ponceau* densitometry. a.u., arbitrary units. * P<0.05 *vs.* CT.

The Exe group presented significantly reduced protein expression levels for KOR compared with the CT group (0.82±0.09 *vs.* 0.46±0.08 a.u.; P<0.05), whereas DOR expression was not different between the groups (0.61±0.12 *vs.* 0.38±0.08 a.u. - Figure 6). The immunoblot bands for DOR and KOR can be found in Figures S1 and S2, respectively. MOR was not detected by Western blotting in the left ventricle in the studied animals.

## Discussion

### Overview of major findings

These experiments provide new and important information regarding the mechanism responsible for exercise-induced cardioprotection. Specifically, our experiments reveal for the first time that the cardioprotective effect of endurance exercise is mediated, at least in part, through activation of the DOR subtype. Our exercise training protocol also increased SOD and catalase activity in the heart but did not increase the left ventricle capillary density, mRNA levels of key antioxidant enzymes or mRNA levels of HSP72. A brief discussion of each of these findings follows.

### Increased myocardial capillary density and HSP72 expression are not involved in exercise-induced cardioprotection

Our results indicated that the left ventricle capillary density did not increase following four independent exercise training sessions. This finding was expected because an exercise-induced remodeling of the vascular network in the heart would likely require several weeks of endurance training [7], [31]. Given that as few as 3–5 consecutive days of exercise have consistently been reported to produce significant cardioprotection, it is clear that the exercise-induced cardioprotection observed in the current experiments was mediated by mechanisms other than increased angiogenesis in the heart [13], [31], [32].

Over-expression of HSP72 in the heart has been suggested to promote cardioprotection against an IR insult [33]. Furthermore, several weeks of endurance exercise training can significantly increase the protein abundance of HSP72 in the heart of rodents [34]. Nonetheless, reports have clearly demonstrated that the prevention of exercise-induced HSP72 expression in the heart does not prevent exercise-induced cardioprotection against IR injury [32], [35]. Therefore, it appears unlikely that an exercise-induced increase in the myocardial levels of HSP72 was responsible for the exercise-induced cardioprotection observed in the current experiments.

### Increased antioxidant enzyme activity may participate in exercise-induced cardioprotection

Similar to Dickson et al. [13], our results indicate that a limited number of exercise sessions did not alter the cardiac gene expression of SOD isoforms and catalase. However, Calvert et al. [11] reported that exercise increased the cardiac expression of CuZnSOD. The apparent discrepancy between our results and those from Calvert et al. seems to be related to differences in the animal model used, the training methodology, and the approach used to evaluate antioxidant activity. In the Calvert et al. study, the protective effects of exercise against myocardial IR injury were investigated by employing voluntary exercise training in mice for 4 weeks, and antioxidant protein expression was analyzed using a Western blotting approach. In our study, the rats were trained at a previously established exercise intensity of ∼70% of VO_2max_ for 4 sessions (less than 1 week), and the cardiac gene expression of CuZnSOD was analyzed via quantitative RT-PCR. Our results revealed that aerobic exercise training increased the MnSOD and catalase activities in the heart. Given that increased myocardial ROS production plays an important role in promoting IR-induced myocardial cell death, it follows that exercise-induced improvements in cardiac antioxidant capacity could be a potential mediator of the cardioprotection observed in the current experiments [36]. Similarly, other independent studies have reported increased MnSOD and catalase activities in the heart following exercise training [11], [36]–[38]. Nonetheless, although exercise promotes an enhanced myocardial antioxidant capacity, it remains controversial whether increased MnSOD levels in the heart are required for exercise-induced cardioprotection. For example, using an antisense oligonucleotide against MnSOD to prevent exercise-induced increases in myocardial MnSOD activity, Yamashita et al. [39] demonstrated that an increase in myocardial MnSOD activity is required to provide training-induced protection against IR-induced myocardial infarction. In contrast, Lennon et al. [40], using the same MnSOD gene silencing approach, reported that prevention of the exercise-induced increase in myocardial MnSOD did not result in a loss of training-induced protection against IR-mediated arrhythmias. Another study concluded that exercise-induced increases in MnSOD contributed to exercise-induced cardioprotection, but other factors must also contribute to this protection [41]. Therefore, based on our results and the work of others [11], [39], [40], improved myocardial antioxidants may contribute to exercise-induced cardioprotection.

### Opioid receptors are involved in exercise-mediated cardioprotection

The current experiments provide evidence that endogenous opioids contribute to the cardioprotective effects of exercise. These findings agree with previous studies that also reported an opioid system-mediated cardioprotective effect of exercise [13], [14], [21]. In this regard, Galvao et al. [21] showed that the effect of chronic exercise training on decreasing IS seems to occur, at least in part, through OR stimulation and not by increasing myocardial perfusion. Concerning the acute effect of exercise-induced cardioprotection, Dickson et al. [13] demonstrated that the administration of a non-selective OR antagonist before exercise abrogated exercise-induced cardioprotection. Our results showed that when trained animals were treated with OR antagonists prior to ischemia, exercise-induced cardioprotection was abolished. It is feasible that the activation of ORs over the course of multiple exercise sessions could initiate a signaling cascade that contributes to the cardioprotective effects of exercise. Taken together, these results underscore the importance of the opioid system, not only as an initial trigger of acute myocardial adaptation to exercise but also as an intermediate pathway contributing to cardioprotection.

### The delta OR is the specific subtype involved in exercise-mediated cardioprotection

To determine which opioid receptor subtype was involved in exercise-induced cardioprotection, we treated exercise-trained animals with specific antagonists prior to exposure to an IR insult. The current study is the first investigation to demonstrate the importance of DOR in exercise-induced cardioprotection. Based on the IPC model of cardioprotection results, we hypothesized that the protective effects of DOR may be linked to the activation of protein kinase C (PKC), which, in turn, opens sarcolemma/mitochondrial K^+^ ATP channels [20], leading to shortening of the cardiac action potential duration by accelerating phase III repolarization [42]. This would inhibit Ca^2+^ entry into the cell via L-type channels and prevent Ca^2+^ overload and the mitochondrial permeability transition pore (MPTP) from opening [7]. The MPTP typically opens during reperfusion, resulting in mitochondrial swelling and protein release, ultimately resulting in necrotic cell death [7]. Nevertheless, further investigations to confirm this sequence of events and to unravel the precise intracellular pathway of DOR-mediated cardioprotection following exercise are required.

We also examined whether exercise training resulted in increased expression of specific ORs in the heart. Our results revealed that exercise did not increase the protein abundance of DOR or KOR in the heart. In fact, exercise training resulted in a significant decrease in the myocardial levels of KOR (and apparently DOR as well). Although the current investigation is the first study to report the impact of exercise training on the protein abundance of specific ORs in the heart, Dickson et al. [13] reported an increase in both KOR and DOR mRNA expression in the heart immediately following exercise. It should be noted however that this increase in cardiac mRNA expression returned to baseline within 8 h following exercise and apparently did not result in increased protein synthesis of these ORs.

The fact that exercise resulted in a decrease in the protein levels of both KOR and DOR in the heart is noteworthy. Specifically, exercise reduced the cardiac protein expression of KOR by ∼44%, whereas DOR expression was diminished by ∼37%. A previous study by Aitchison et al. [43] demonstrated that activation of KOR signaling promotes an anti-cardioprotective state, resulting in an increased IR-induced IS in the rat heart compared with controls. Therefore, the exercise-induced reduction in cardiac levels of KOR may serve as a cardioprotective adaptation. Indeed, the opposing outcomes of DOR and KOR stimulation could establish a balance in the opioid signaling system. Therefore, exercise-induced cardioprotection could be produced not only through DOR stimulation but also through KOR downregulation. Interestingly, KOR and DOR have been consistently implicated in IPC- and remote IPC-induced cardioprotection [15], [20], [44]–[52], reinforcing a potential common cardioprotective link between exercise, ORs and IPC. Exercise may induce cardioprotection via DOR and KOR in a signal transduction pathway similar to IPC-induced cardioprotection. However, further research describing the signaling pathways of DOR and KOR and their interactions is required to provide additional insight regarding the OR mechanism(s) of exercise-induced cardioprotection.

Finally, MOR expression in the heart was also investigated in the present study. Nonetheless, using a Western blot approach, there were no detectable levels of MOR protein in the heart. This finding agrees with previous studies that also did not detect MOR expression in the rat heart [43], [53], [54]. Together, these findings suggest that MOR do not play a role in the modulation of IS.

### Study limitations

The results of the present study should be interpreted while considering certain limitations, particularly aspects related to methodology and protocol. First, the best method for measuring myocardial IS, as well as the use of TTC as the single approach to quantitatively estimate myocardial injury, is still extensively debated in the literature. TTC is an inexpensive, rapid, and reliable method for myocardial IS measurement and has been used as the sole quantitative method in several investigations involving IS assessment [13], [39], [55], [56]. Moreover, TTC has been extensively utilized as the gold standard technique to validate new imaging methods of infarct area quantification, such as cardiac magnetic resonance imaging [57]–[61], contrast echocardiography [62]–[66], and two-dimensional speckle tracking imaging [67]. Nevertheless, the use of biochemical techniques, such as a dosage of troponin-I, may also be reliable for estimating myocardial IS and could confirm and emphasize our results concerning the effects of OR antagonists in exercise-induced cardioprotection. Second, the use of a single fixed dose of antagonists is always a problem in pharmacological testing. In our study, based on the large dose of OR antagonists used, the low density of ORs in cardiac tissue, previous results from Schultz et al. [17], [44] and preliminary data from our lab, we assumed that 3 mg/kg of OR antagonists would be the appropriate dose to demonstrate the involvement of the opioid system in exercise-induced cardioprotection. Although the ideal protocol would involve establishing a dose-response curve for all antagonists, ethical concerns related to the large number of animals used in the study and the increase in operational costs limited the feasibility of more experiments. Third, the effect of OR antagonists on antioxidant enzyme activity is a very interesting issue that was not addressed in the present study. Indeed, increased myocardial antioxidant capacity is considered an important mechanism involved in exercise-induced cardioprotection, and OR antagonists may theoretically interfere with enzyme expression. However, no evidence exists that any of the OR antagonists used in the current experiments are capable of inhibiting antioxidant enzyme activity. Further, it appears unlikely that any of these OR blockers are capable of crossing the mitochondrial inner membrane to inhibit MnSOD, the only antioxidant enzyme that has consistently been shown to be important in exercise-induced cardioprotection [68]–[70]. Nevertheless, further studies are warranted to better determine the effects of OR antagonists on antioxidant enzyme activities.

### Summary and Conclusions

Although it is well established that regular bouts of endurance exercise result in cardioprotection against IR injury, the mechanisms responsible for this cardioprotection remain under debate. Although an increase in myocardial antioxidants (e.g., MnSOD) may contribute to exercise-induced cardioprotection, the current experiments demonstrate that endogenous opioids also contribute to exercise-induced cardioprotection. Importantly, our results reveal for the first time that the DOR subtype plays an essential role in exercise-induced cardioprotection. Indeed, pharmacological blockade of DOR resulted in a significant loss of exercise-induced cardioprotection against IR-induced injury. However, additional experiments are required to determine if a significant interaction exists between exercise-induced changes in DOR signaling and increased myocardial antioxidants in the heart.

## Supporting Information

Figure S1 (TIF)

Figure S2 (TIF)
