# Supplementary figures and images for: Delta Opioid Receptors: The Link between Exercise and Cardioprotection
Source: PLoS One. 2014 Nov 21;9(11):e113541. doi: 10.1371/journal.pone.0113541 (PMC4240613; doi:10.1371/journal.pone.0113541)

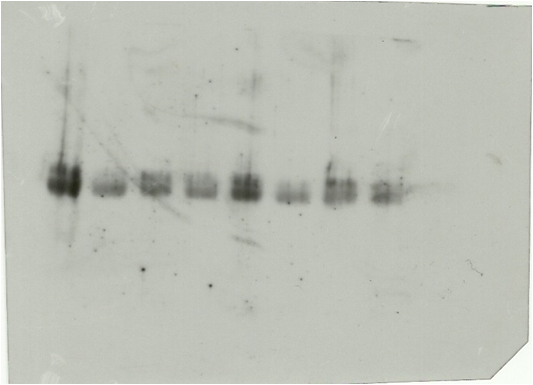

Supplement: Figure S1 — (TIF) [file pone.0113541.s001.tif]

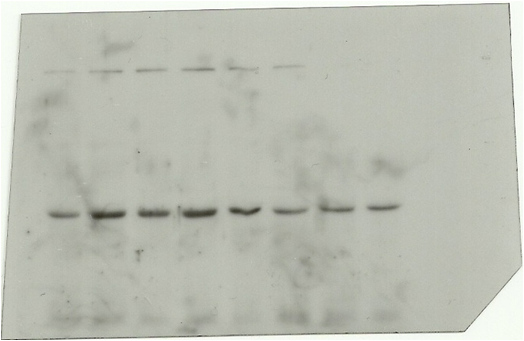

Supplement: Figure S2 — (TIF) [file pone.0113541.s002.tif]
